# H1N1 Influenza Viral Infection in a Postpartum Young Woman Causes Respiratory Failure: What the Care Providers Ought to Know?

**DOI:** 10.1155/2012/419528

**Published:** 2012-10-23

**Authors:** Stavros Aloizos, Paraskevi Aravosita, Christina Mystakelli, Efthymia Kanna, Stavros Gourgiotis

**Affiliations:** Intensive Care Unit, Mitera Obstetrics/Gynecology clinic, 15669 Athens, Greece

## Abstract

Pregnant and postpartum women are considered a population at increased risk of hospitalization of H1N1 infection. We report the case of a young postpartum woman, who developed evidence of respiratory failure reaching the point of requiring intubation due to an H1N1 influenza virus infection two days after a caesarean delivery. We emphasize the diagnosis, management, and the outcome focusing on the question “what the care providers, including obstetric health care workers, ought to know?” Diagnostic and management strategy for pregnant or postpartum women with novel influenza A (H1N1) viral infection and increased awareness amongst patients and health care professionals may result in improved survival.

## 1. Introduction

Since 2009, the infection with the novel H1N1 influenza virus, popularly termed “swine flu,” prompted the World Health Organization (WHO) to state that during pregnancy both mother and baby are at increased risk when infected with either pandemic or seasonal influenza and that pregnant women should be vaccinated [1]. A reported systematic literature review found that pregnancy was associated with increased risk of hospital and intensive care unit (ICU) admission and death, while pregnant women who received delayed treatment with neuraminidase inhibitors or who had additional risk factors were more likely to develop severe disease and preterm births [2].

We report the case of a young postpartum caucasian woman, with no preexisting illness, presenting with respiratory manifestations of H1N1 influenza virus infection two days after Caesarean delivery of a healthy newborn. The patient developed evidence of respiratory failure reaching the point of requiring intubation and a long time intensive care management.

## 2. Case Presentation

A 30-year-old postpartum woman reported cough, shortness of breath, myalgia, and fever until 38.3°C. The patient had an uneventful cesarean delivery two days ago. She had been in good health throughout her pregnancy and had not traveled abroad or been exposed to anyone with confirmed or probable seasonal or novel influenza. However, she reported a 2-day history of rhinorrhea, cough, and temperature until 37.4°C, before her delivery. She denied nausea, vomiting, diarrhea, or abdominal pain. She had no chronic medical problems, did not smoke or use illicit drugs, and rarely drank alcohol. She also was hesitant to receive both the H1N1 and influenza vaccines during her pregnancy.

**Figure 1:**
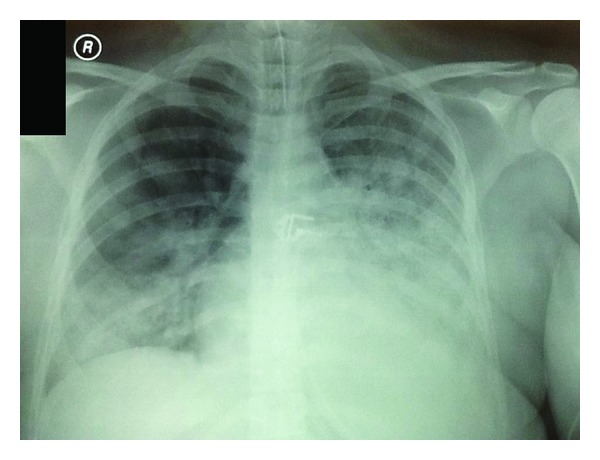
Chest radiograph of day 1 showing bilateral alveolar infiltrates and a dense area of consolidation with a small pleural reaction in the left hilar region.

Initial vital signs included BP: 110/55 mmHg, HR: 114 beats/minute, and SpO_2_: 95% in room air, while she was tachypneic at 20 breaths/min. Arterial blood gas analysis demonstrated pH: 7.44, PaO_2_: 90 mmHg, and PaCO_2_: 32 mmHg. Results of the initial laboratory analysis included WBC: 12,430 cells/*μ*L, neutrophils: 88%, lymphocytes: 6%, CRP: 10.9 mg/dL, SGOT: 48 U/L, ALKP: 139 U/L, and *γ*-GT: 201 U/L. Her physical examination was notable for decreased breath sounds, rhonchi, and wheezing in bilateral lung fields. The initial chest radiograph revealed bilateral alveolar infiltrates and a dense area of consolidation with a small pleural reaction in the left hilar region (Figure 1).

Due to the fact that community-acquired pneumonia was suspected, a broad-spectrum antibiotic coverage was initiated, with the support of oxygen therapy and bronchodilators. At the same time, urine sample was sent for possible detection of pneumoococcus and legionella antigens. A rapid influenza diagnostic antigen test (RIDT) was negative.

**Figure 2:**
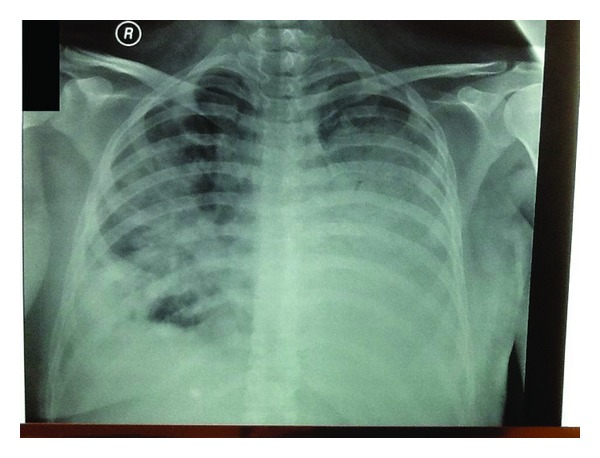
Chest radiograph of day 2 showing diffuse alveolar opacities extending to the left upper lung fields, but in the right middle and lower lobe.

During the first 24 hours after her admission in the ICU, the patient's respiratory status continued to deteriorate. An arterial blood gas revealed pH: 7.41, PCO_2_: 23 mmHg, and PO_2_: 63 mmHg, while receiving 100% FiO_2_ via non-rebreather mask. There was also a characteristic radiological burden on chest radiograph with diffuse alveolar opacities extending to the left upper lung fields, but in the right middle and lower lobe (Figure 2).

**Figure 3:**
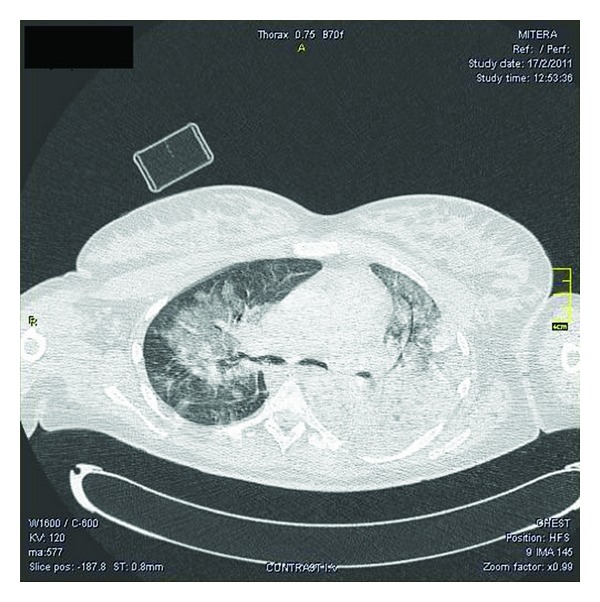
Chest CT confirming bilateral basilar pulmonary consolidation, consistent with pneumonic infiltrates.

The decision was made to intubate the patient for impending respiratory failure. Subsequent real-time reverse transcription-polymerase chain reaction (RT-PCR) analysis of a nasal swab specimen for influenza subtyping confirmed a diagnosis of novel influenza A (H1N1) infection. She was started on oseltamivir and broad-spectrum antibiotics for possible secondary infection. Corticosteroids were provided while the patient's hypoxemia stabilized with a trial of airway pressure release ventilation. A chest computed tomography (CT) confirmed bilateral basilar pulmonary consolidation, consistent with pneumonic infiltrates (Figure 3).

During her hospitalization, the patient remained hemodynamically stable with intensive care management without major complications or phenomena of other organs' failure. However, she sometimes showed worsening of her ventilation due to partial atelectasis. To resolve the problem of obstructive events, we performed three urgently bronchoscopies. In addition, Acinetobacter baumannii was isolated from the bronchial secretions (ventilator-associated pneumonia; VAP) and a new intravenous antibiotic scheme was administrated based on her bronchial cultures susceptibility.

The patient was weaned from mechanical ventilation on day 15 and transferred to the obstetrical floor the following days. She continued to recover uneventfully and was discharged with her healthy newborn on day 20. The 4-month followup with chest CT continued to be abnormal with areas of pulmonary fibrosis while spirometry findings had features of restrictive syndrome.

## 3. Discussion

The risks of morbidity and mortality from seasonal and pandemic influenza H1N1 are now known to be greater in pregnant than in nonpregnant and postpartum women, especially pregnant women in the 3rd trimester [3]. In one study, the authors estimated that the relative risk of hospitalization, admission to ICU, and death was 5.2, 6.5, and 1.4, respectively, for pregnant women [4].

Gestational age is associated with higher risk of developing critical infection; the risk increases with the weeks of gestation while women in the 2nd or 3rd trimester of pregnancy have a higher rate of developing critical infection [5]. It may be related to specific immune suppression, decreased resistance, and physiological changes in pregnancy.

The patients may present with symptoms for influenza-like illnesses (fever, malaise, cough, sore throat, rhinorrhea, headache, myalgia, vomiting, and diarrhea) and they may demonstrate significant abnormalities on complete blood counts and chest radiographs [6]. The median time from onset of symptoms to hospitalization ranges from 2 to 6 days.

RIDT detects influenza viral nucleoprotein antigen and is used as screening diagnostic tool. This test often provides results in 30 minutes or less; however, it has low sensitivity (10% to 70% for H1N1, while a negative test does not rule out influenza) and cannot distinguish between virus subtypes; so a specific diagnosis of influenza A (H1N1) cannot be established [7]. RT-PCR assays are the most sensitive (86%–100%) and specific tests available, but generally require 48 to 96 hours to process [7]. Confirmatory tests include RT-PCR and viral cultures on properly collected upper respiratory tract specimens. Although the isolation of H1N1 virus by culture is the “gold standard” diagnostic test, the procedure typically is too slow to guide clinical management [8].

Early admission to the ICU for respiratory support may be required. Treatment of severe influenza A (H1N1) is primarily supportive, although a role for antiviral medications exists. Currently, the CDC recommends treatment of all hospitalized patients with suspected, probable, or confirmed A (H1N1) or seasonal influenza with either oseltamivir or zanamivir [9]. Treatment with oseltamivir should commence as soon as possible and antiviral treatment should be provided even if started later than 48 h. The recommended duration of therapy is 5 days, although a longer course can be considered in severe cases. Also of note, new mothers should be considered as high risk and treated as such until 2 weeks postpartum.

About 1% to 10% of patients with clinical illness due to the novel infection have required hospitalization and the overall case fatality ratio has been estimated as <0.5%. [10] Rapidly progressive respiratory failure is relatively common and about 10% to 30% of hospitalized patients have required ICU admission [10]. In the present case, we initially tried to stabilize the patient's hypoxemia using noninvasive positive pressure ventilation (NPPV). There was no hemodynamic instability and no multiorgan, thus, there were no contraindications to application of NPPV. Unfortunately, we observed no spectacular results; NPPV temporarily improved oxygenation and reduced the work of breathing, but did not alter the course of the disease. On the other hand, NPPV is a procedure in which there is possibly increased risk of respiratory pathogen transmission [11]. Our patient, in a few hours, required invasive mechanical ventilation (IMV) support. IMV, with a lung-protective ventilation strategy, is recommended as the appropriate approach for managing patients with pandemic A (H1N1) infection complicated by respiratory failure [10].

Low-dose systemic steroids may be considered for patients with refractory septic shock [10]. However, early use of corticosteroids might prolong viral replication in severe acute respiratory syndrome (SARS), while during the SARS period in the ICU, the use of systemic steroids is associated with an increase rate of MRSA, Stenotrophomonas, and candida species acquisition, and ventilator-associated pneumonia [12].

**Figure 4:**
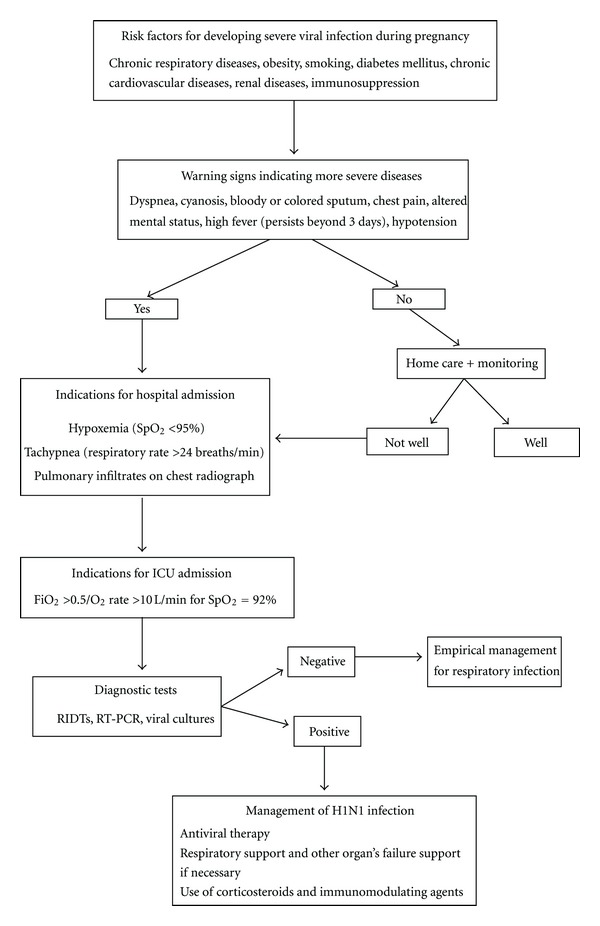
Diagnostic and management strategy for pregnant or postpartum women with novel influenza A (H1N1) viral infection (ICU: intensive care unit; RIDTs: rapid influenza diagnostic antigen tests; RT-PCR: real-time reverse transcription-polymerase chain reaction; CT: computed tomography; SpO_2_ = oxygen saturation).

In Figure 4, a diagnostic and treatment strategy of pregnant and postpartum women with influenza A (H1N1) virus infection is encompassed. Specifically, risk factors for severe disease include pregnant women in their 2nd or 3rd trimester of pregnancy during the influenza season and women at any stage of pregnancy with certain chronic medical conditions. Patients should be referred for ICU assessment if FiO_2_ of >0.5 or oxygen at a rate of <10 L/min is required to maintain the SpO_2_ at 92%.

All hospitalized patients with suspected influenza should be tested. Nasal swabs with nasal secretions or nasopharyngeal aspirates or swabs are appropriate specimens for detecting human influenza A. RT-PCR or viral culture should be mainly performed as confirmatory testing. Patients with illnesses compatible with pandemic A (H1N1) virus infection but with negative RIDT results should be treated empirically based on the level of clinical suspicion, underlying medical conditions, severity of illness, and risk for complications.

Inactivated influenza vaccine can be safely and effectively administered during the 2nd and 3rd trimester. No study has demonstrated an increased risk of either maternal complications or adverse fetal outcomes or adverse events among children born to women who received inactivated influenza vaccine during pregnancy [13].

Finally, consideration of the health of the fetus is an additional concern. Clinicians should attempt to maintain a minimum PaO_2_ of 70 mm Hg to ensure adequate fetal oxygenation. The fetus is better supported in uterus up to 32–34 weeks of gestational age, if the mother can maintain adequate hemodynamics, oxygenation, and ventilation.

In conclusion, this case of H1N1 infection in relatively normal postpartum woman illustrates the increased risk of life threatening complications in this group. Thus, increased awareness amongst patients and health care professionals and a higher uptake of prevention strategies may result in improved survival in future epidemics. It is critical that all health care providers use proper preventive measures to avoid infection and appropriately manage those who are affected.
